# A mapping and gapping analysis of patient and public involvement in a clinical trials unit

**DOI:** 10.1186/1745-6215-14-S1-P93

**Published:** 2013-11-29

**Authors:** Heather Bagley, Paula Williamson, Carrol Gamble

**Affiliations:** 1 Clinical Trials Research Centre, University of Liverpool, Liverpool, Merseyside, UK

## 

Patient and public involvement (PPI) in research involves the active contribution of members of the public in research studies and within research organisations. PPI is important in ensuring the relevance and quality of general health care research. Funders often now request evidence of prior and future planned PPI in health research.

The University of Liverpool Clinical Trials Research Centre recently appointed a part time Patient and Public Involvement Co-ordinator. In order to inform the development of a PPI strategy for the Unit, the Co-ordinator is conducting a mapping and gapping exercise to identify where, when and how previous and current trials involve Public Research Partners (PRPs) and where there is potential to increase and improve such involvement. This analysis will seek to:

• Map the amount, type and stages of PPI taking place in existing trials in the Unit, including Research Network involvement

• Describe current processes for recruiting, training, support and funding of Public Research Partners in the CTRC

• Identify current mechanisms for evaluating and documenting the impact of PPI in trials run by the CTRC

• Indicate current gaps in PPI within the CTRC and propose recommendations to address these gaps.

The facilitator will conduct semi structured interviews with trial co-ordinators and will review PPI plans of trials being run by the unit to examine both involvement in the research application process and to examine what was planned in terms of PPI at the outset. The results from this mapping and gapping exercise will be presented.

